# Clinical-pathological issues in thyroid pathology: study on the routine application of NIFTP diagnostic criteria

**DOI:** 10.1038/s41598-019-49851-1

**Published:** 2019-09-12

**Authors:** Valentina Canini, Davide Leni, Angela Ida Pincelli, Marcella Scardilli, Mattia Garancini, Chiara Villa, Camillo Di Bella, Giulia Capitoli, Riccardo Cimini, Biagio Eugenio Leone, Fabio Pagni

**Affiliations:** 1 Pathology, Department of Medicine and Surgery, University of Milano-Bicocca, San Gerardo, Italy; 2 Radiology, Department of Medicine and Surgery, University of Milano-Bicocca, San Gerardo, Italy; 3 Department of Endocrinology, San Gerardo Hospital, Monza, Italy; 4 Department of Surgery, San Gerardo Hospital, Monza, Italy; 5 Department of Otolaryngology, San Gerardo Hospital, Monza, Italy; 6 Center of Biostatistics for Clinical Epidemiology, Department of Medicine and Surgery, University of Milano-Bicocca, Vedano al Lambro, Italy

**Keywords:** Cell biology, Biological techniques

## Abstract

In 2017, the WHO classification of tumours of the endocrine organs established the criteria for a NIFTP diagnosis. The present paper considers some aspects that are still debated or unresolved: the real incidence and clinical meaning of multifocal/multinodular lesions, the biological behaviour of micro-NIFTP, the sprinkling phenomenon and the corresponding modifications to the FNA reporting systems based on changes to the ROM. Moreover, the paper suggests possible scenarios for the clinical-pathological management of this entity. From the initial 1470 cases, a group of 68 NIFTPs was recruited in a 9 year-long period. The average age at diagnosis was 55 years. The average diameter of the lesion was 1.7 cm (0.1 cm–10 cm). In 41 cases (60.1%), the lesion was inserted in the context of a multinodular background. In 12 cases, the diagnosis was incidental and the pre- operative FNA was performed on a different target. In 10 out of 68 cases, there was a multifocal NIFTP; in 14.7% of patients, PTC-like nuclear features showed sprinkling phenomenon. The cytological revision allocated 21 cases (49%) to the SIAPEC TIR3 indeterminate class and a nuclear score 2 or 3 were identified in 25 smears. Multifocality is part of the spectrum of NIFTPs, that can arise in a multinodular background with variable sizes from microscopic lesions to very large ones. Cytopathological criteria such as an evaluation of the nuclear score may help the pathologists in promoting a NIFTP diagnosis in the preoperative setting.

## Introduction

The IV WHO classification of tumours of the endocrine organs in 2017 abolished the old diagnostic category of *non-invasive encapsulated follicular variant* Papillary Thyroid Carcinomas (EFVPTC), that was replaced by the new terminology Noninvasive follicular thyroid neoplasms with papillary-like nuclear features (NIFTP)^1,2^. The diagnosis of NIFTP is performed only on surgical specimens and takes into account stringent histological criteria^1,2^. NIFTP should have a well-defined capsule, clearly separated from the surrounding parenchyma and without infiltration foci. The architectural pattern of the lesion is follicular, rendering it necessary to exclude the presence of true papillae. These kinds of tumours have nuclear characteristics that are similar to those of classical Papillary Thyroid Carcinoma (PTC). Despite the great utility related with the introduction of a more accurate terminology to define these indolent (pre-malignant) lesions, different issues remain unresolved as of today. Firstly, a minimum (or maximum) size-limit of NIFTP is not defined, although the majority of the studies focused on lesions with dimensions of 1 cm or more^3–5^. Furthermore, no obvious data is available for bilateral (or multifocal) NIFTP lesions^5–9^. Another debated aspect regards the extent of the Papillary Thyroid Carcinoma-like (PTC-like) nuclear alterations necessary for the diagnosis. In particular, the so-called *sprinkling* phenomenon (heterogeneity of the PTC-like nuclear features within the same lesion) may be encountered in the routine practice with relevant diagnostic consequences^10^. Finally, fine needle aspiration (FNA) reporting systems are introducing some changes to the risk of malignancy (ROM) percentages of indeterminate classes, related with the NIFTP advent^3,7,11–16^. The present paper on a retrospective NIFTP series addresses the highlighted issues and suggests possible scenarios for the clinical-pathological management of this entity.

## Materials and Methods

### Histopathology

From a retrospective series of thyroid surgical specimens (N = 1470), recruited at the Department of Pathology, San Gerardo Hospital, University of Milano-Bicocca, Monza, Italy since January 1^st^ 2010, 84 cases with diagnosis of EFVPTC (up to December 2016) and 22 NIFTP (since January 2017 to December 2018) were extracted. The original formalin-fixed paraffin-embedded (FFPE) and haematoxylin and eosin stained histological sections were re-evaluated according to the classification criteria proposed by Nikiforov *et al*. then introduced into the WHO classification of tumours of the endocrine organs of 2017, 4th edition, by a panel of expert thyroid pathologists (FP,CDB,BEL)^1,2^. In particular, the following parameters were used as inclusion criteria.

**Figure 1:**
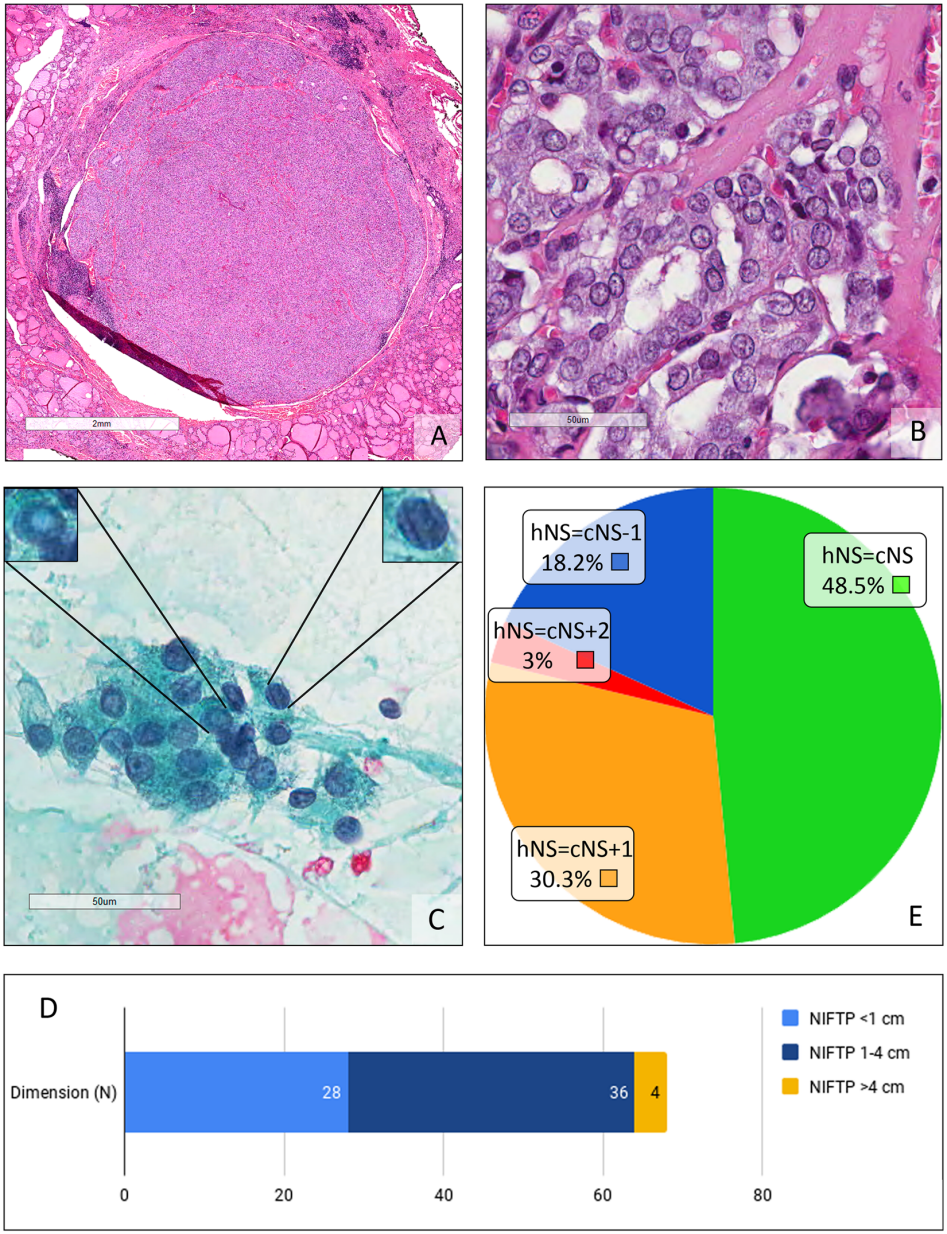
(**A**) Micro Noninvasive follicular thyroid neoplasm with papillary-like nuclear features (NIFTP). This incidental, 5 mm diameter lesion is completely encapsulated. (H&E, 2x). (**B**) Detail on cells of the micro Noninvasive follicular thyroid neoplasm with papillary-like nuclear features (NIFTP). Regarding size and shape, membrane irregularities and chromatin characteristics resulting in a NS of 3. (H&E, 40x). (**C**) Cytological smear of a Noninvasive follicular thyroid neoplasm with papillary-like nuclear features (NIFTP). On the left, magnification of a cell with a pseudoinclusion. On the right, magnification of a cell showing grooves. The final diagnostic class was THY4-suspicious of malignancy. (Papanicolau, 40x). (**D**) Distribution of the diameters in our Noninvasive follicular thyroid neoplasm with papillary-like nuclear features (NIFTP) case series. (**E**) Comparison between the cytological nuclear score (cNS) and histological nuclear score (hNS).
- Follicular growth pattern, with absence of papillae, absence of psammomatous bodies, solid/trabecular/insular growth pattern <30%, absence of necrosis and low mitotic index (less than 3 mitoses per 10 high power fields);
- PTC-like nuclear characteristics, with nuclear score (NS) of 2 or 3 (Fig. 1B). The nuclear score included the evaluation of the size, shape and clarification of the tumour nuclei, as previously described^1,2^.
- Negative immunohistochemical BRAF ve staining (Spring Bioscience, Pleasanton, CA, USA with an Extend Anti-Rabbit Polymer HRP amplification kit Dako, Glostrup, Denmark) with confirmation of the wild type BRAF status by reverse hybrid allele specific oligonucleotide hybridization (ViennaLab BRAF StripAssay, Vienna, Austria).

- Presence of capsule, thin or thick, or clear demarcation of the lesion with respect to the surrounding parenchyma and absence of vascular or capsular invasion (Fig. 1A);

### Clinical features

For the final clinical-pathological characterisation of the NIFTP series, the following parameters were recorded: sex of the patient; age at diagnosis; maximum tumor diameter; presence/absence of lymph node metastasis at presentation; mean, median and range of follow-up in years; uninodular/multinodular background (goiter, chronic lymphocytic thyroiditis, concurrent thyroid tumours); *incidental* tumor; single/multifocal lesion; NIFTP showing “sprinkling” phenomenon. NIFTP was considered “multifocal” if two or more lesion foci were present in the same thyroid lobe or in both lobes. In the case of multifocal lesions, the maximum diameter was evaluated taking into account the lesion with the largest diameter. NIFTP was considered *incidental* if the preoperative fine needle aspiration (FNA) was performed on a different target.

### Cytopathology

The original cytological preparations that referred to the preoperative FNAs were retrieved. Traditional Pap-stained smears were diagnosed according to the 5-tiered 2014 Italian SIAPEC-IAP classification system, which is comparable to UK Royal College of Pathology system (Fig. 1C)^17–20^. To confirm the representativity of the series, the incidence of the different diagnostic class was compared to a standard, expected, distribution taken from the study of Bizzarro *et al*.^21^ and to a target distribution of the international review by Bongiovanni *et al*.^16^. The cytological NIFTP NS was also calculated in blind from the histological results.

### Statistical analyses

Statistical analyses were performed using the open-source R software v. 3.5.0. A linear regression approach was used to evaluate the impact of diameters on an explanatory variable. To compare the evaluated NS on cytological and histological samples, and to compare the diagnostic classes and distribution of our data with an italian cohort population and a target distribution of an international review, the exact Fisher test was used. For each test, the level of significance was set equal to 0.05.

All methods of the study were carried out in accordance with relevant guidelines and regulations; the protocol was approved by a ASST Monza Ethical Board (AIRC MFAG 2016 Id. 18445, HSG Ethical Board Committee approval October 2016, 27102016). Appropriate informed consent was obtained from all subjects of the study.

## Results

**Figure 2:**
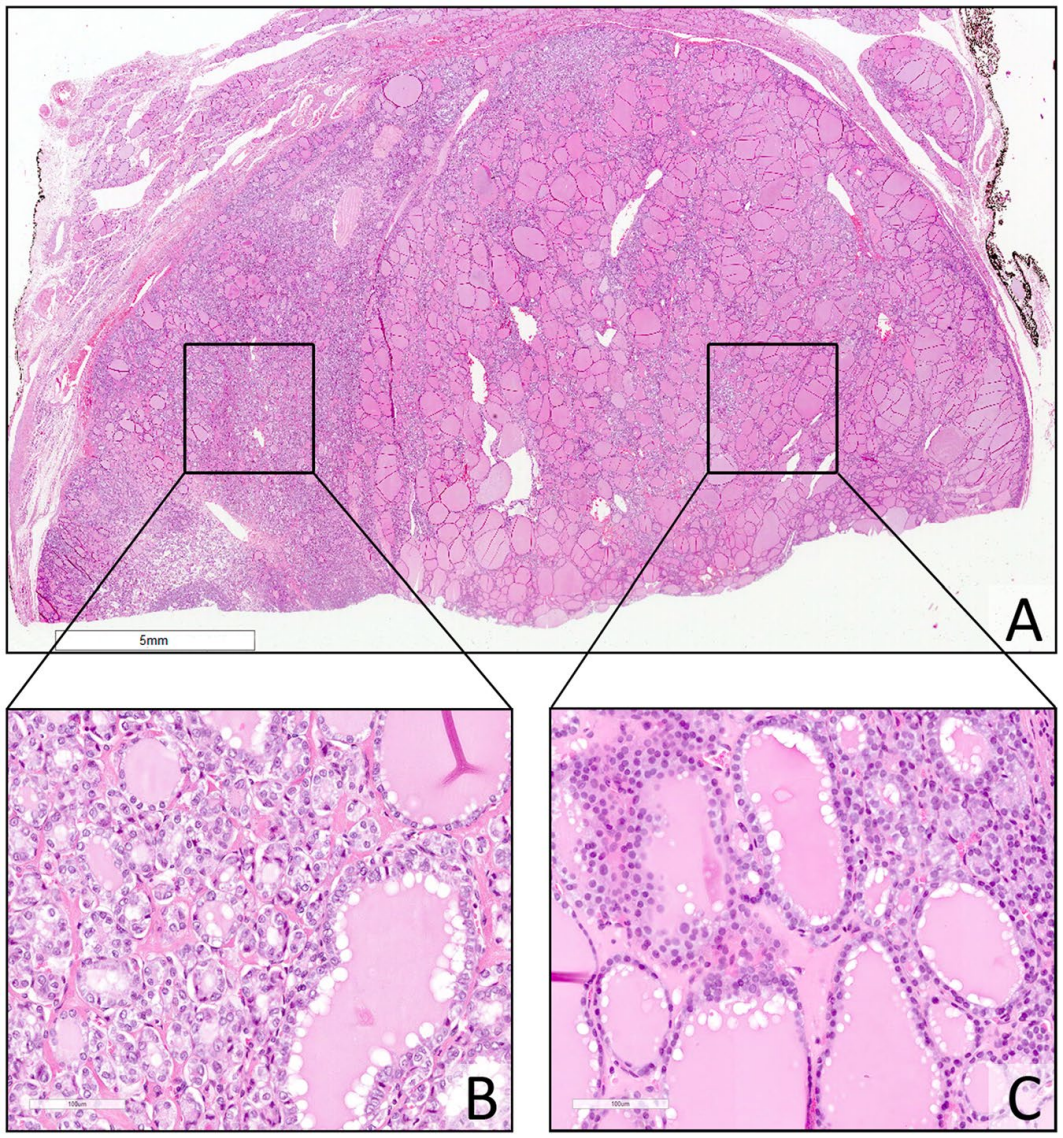
(**A**) Sprinkling Noninvasive follicular thyroid neoplasm with papillary-like nuclear features (NIFTP). (H&E, 4x). (**B**,**C**) Different cells populations Noninvasive follicular thyroid neoplasm with papillary-like nuclear features (NIFTP) showing the sprinkling; on the left, papillary thyroid carcinoma-like (PTC-like) features and, on the right, follicles with dark, hyperplastic-like nuclei. (H&E, 20x)

From the EFVPTC group, 7 cases were excluded due to the unavailability of the BRAF status. Of the remaining 77 cases:

- 17 cases were reclassified as *invasive* EFVPTC due to capsular invasion being clearly present;
- 10 cases were reclassified as well-differentiated tumours of uncertain malignant potential (WDT-UMP, WHO 2017) because capsular /vascular invasion was doubtful;
- 4 cases showed focal papillae^22^;

Overall, 46 out of 77 cases (59.7%) were reclassified as NIFTP due to satisfying all the required diagnostic criteria. Therefore, the study consisted of a group of 68 NIFTPs in a 9 year-long period (50 female patients −74%). The average age at diagnosis was 55 years (range 21–81 years). The average diameter of the NIFTP at diagnosis was 1.7 cm (range between 0.1 cm and 10 cm, standard deviation 1.6, median 1.3 cm). In 28 out 68 cases (41%), the diameter was <1 cm; while in 4 patients >4 cm.(Fig. 1D). The FNA diagnostic class was available in 55 out of 68 cases (80.8%). Table 1 compares the distribution of the most relevant clinical-pathological variables in the general population having a thyroid nodule^22–24^, in NIFTP Nikiforov’s study^22–24^ and in our series. The average age of the patients included in our series was approximately 10 years greater than that of the Nikiforov series, whilst it is slightly lower than the average age of the general population with a thyroid nodule. The average size of our NIFTPs does not differ from the general population, whilst it is lower than observed in Nikiforov’s series in which it can be noted that lesions smaller than 1 cm in diameter were not considered. Lymph node metastasis at presentation was absent in all cases (Table 2). The mean time of follow-up was 42 months (median 48 months, range 1–96 months). In this period no patient encountered recurrence. In 41 cases (60.1%), the lesion was inserted in the context of a multinodular background (mainly goiter, Table 2). In 5 cases, NIFTP was associated with a cPTC (3 of them were micro PTC) and, in 1 case, NIFTP was associated with a follicular tumour of uncertain malignant potential (ft-UMP). The FNA report was available in 32 of them; in 12 cases the diagnosis of NIFTP was *incidental* and the pre-operative FNA was performed on a different target (Table 2). The difference between the diameter of the 43 cases with FNA performed on target on the NIFTP (mean 1.93 cm) and the NIFTP diameter in these 12 cases (mean 0.69 cm) was investigated through a linear regression model and a negative association was found between class membership and thyroid nodule diameters. A statistically significant relationship remained when controlling for age and gender (β = −0.85, p-value = 0.039). In 10 out of 68 cases (14.7%), there was a multifocal NIFTP; 1 of them was unilateral (left thyroid lobe) whilst in 2 cases pathologists highlighted the simultaneous involvement of a thyroid lobe and isthmus (Table 2). At the histological re-examination, in 10 out of 68 cases (14.7%), PTC-like nuclear features showed “sprinkling” phenomenon (Table 2, Fig. 2). Excluding 12 FNAs that were out of target, the cytological revision allocated 21 cases to the cytological diagnostic category TIR3 (indeterminate, 49%); 14 cases (32%) were TIR4 (suspicious for malignancy); 6 cases (14%) were in the cytological diagnostic category TIR2 (benign); 2 cases (5%) were included in the cytological diagnostic category TIR5 (malignant)^17–20^. The distribution of our 43 cases reflected the pivotal studies of Bizzarro *et al*. and Bongiovanni *et al*. without a statistically significant difference (p-value equal to 0.749 and 0.567 respectively)^16,21^ (Table 3).

**Table 1:** Comparison between our study on Noninvasive follicular thyroid neoplasm with papillary-like nuclear features (NIFTP), Nikiforov *et al*. study on encapsulated follicular variant of Papillary Thyroid carcinoma (EFVPTC) and data of a general population with thyroid nodules, including age, sex and lesion diameter. (Age and lesion diameter are reported as mean with range value in brackets)^2,22–24^.

	Current study on 68 NIFTP	Nikiforov’s study on 109 EFVPTC	General population with thyroid nodules
Age, years	55 (21–81)	45.9 (21–81)	>60
Sex F M	N (%) 50 (74) 18 (26)	N (%) 91 (83) 18 (17)	>F <M
Lesion diameter, cm	1.7 (0.1–10)	3.1 (1.1–9.0)	1.5

**Table 2:** Lymph node metastasis at presentation, multinodular background Noninvasive follicular thyroid neoplasm with papillary-like nuclear features (NIFTP), incidental NIFTP, multinodular NIFTP, sprinkling NIFTP in our case serie.

	Lymph node metastasis at presentation (N)	Multinodular background (N)	Incidental finding (N)	Multifocal NIFTP (N)	Sprinkling NIFTP (N)
Yes	0	41	12	10	10
No	68	27	56	58	58
Total	68	68	68	68	68

**Table 3:** Comparison between the distribution of Noninvasive follicular thyroid neoplasm with papillary-like nuclear features (NIFTP) in the cytological diagnostic classes according to SIAPEC-IAP 2014 system in our series, in Bizzarro *et al*. study and data from Bongiovanni *et al*. review^16,17,21^.

	Current study N (%)	Bizzarro *et al*. study N (%)	Bongiovanni *et al*. study (%)
TIR 1/THY1/Non diagnostic	0 (0%)	0 (0%)	(3%)
TIR 2/THY2/Benign	6 (14%)	5 (13.5%)	(10%)
TIR 3/THY3a - THY3f/AUS/FLUS - FN/SFN	21 (49%)	15 (40.6%)	(51%)
TIR 4/THY4/Suspicious for Malignancy	14 (32%)	13 (35.1%)	(24%)
TIR 5/THY5/Malignant	2 (5%)	4 (10.8%)	(8%)
Total cases	43 (100%)	37 (100%)	(100%)

The *cytological* NS was calculated in 33 out of the 43 cases in which the FNA was performed appropriately on target because in 10 cases the morphological re-evaluation of the original slides was not available (Table 4, Fig. 1E).

- 8 cases had cytological NS = 1;
- 12 cases had cytological NS = 2;
- 13 cases had cytological NS = 3.
  Of the same 33 cases, the *histological* NS was calculated on FFPE sections:
- 22 cases had histological NS = 2;
- 11 cases had histological NS = 3.

**Table 4:** Comparison between nuclear score evaluated on cytological smear and nuclear score evaluated on histological fomalin-fixed paraffin-embedded (FFPE) sections.

	score 1 (N)	score 2(N)	score 3(N)
NS cytology	8	12	13
NS histology	/	22	11

Abbreviations: NS = nuclear score.

By comparing the number of cases with a different NS, evaluated on cytological smears and histological sections, a statistically significant difference was found when the exact Fisher test was performed (p-value 0.002).

## Discussion

Despite the indolent nature of the *non-invasive* EFVPTC, the word “carcinoma” included in its name has nevertheless led clinicians to perform an aggressive treatment (total thyroidectomy and radioiodine) for many years^2^. In 2016, an international group of pathologists proposed that the term *non-invasive* EFVPTC should be replaced with NIFTP, outlining the morphological inclusion criteria necessary to make this diagnosis^2^. The new terminology has been accepted and introduced in the 2017 WHO Classification of tumours of the endocrine organs^1^. In our study, we analysed a retrospective cohort of patients with a previously established diagnosis of EFVPTC and patients recruited after 2016 with a diagnosis of NIFTP. A team of thyroid-committed pathologists performed the morphological revision of the histological series to obtain a final homogeneous group of 68 cases. This was followed by the correlation with the preoperative cytological SIAPEC-IAP diagnostic class and the pathological re-evaluation of the original FNAs whenever available.

Among the original EFVPTCs, NIFTPs accounted for 59.7% of cases. In a previous study by Pagni *et al*. that focused on a PTC population that contained 287 cased observed in an 8 year-long period, in the same routine practice, the follicular variant of PTC accounted for the 40% of the cases (115 out of 287)^25^. Due to the clearcut evidence of capsular invasion, the former *carcinoma* diagnosis was confirmed in a significant rate of patients (22%), while 10 out of 77 EFVPTCs were reclassified as WDT-UMP because capsular or vascular invasion was doubtful. In those situations, although the application of the morphological criteria was rigorous, doubts persisted especially with respect to the architectural criterion “*complete capsule /distinct separation of the lesion from the surrounding parenchyma”* because of initial/incomplete penetration phenomena (mushrooms). The board reconfirmed that a correct sampling of the lesion is essential for a complete morphological evaluation along with a scrupulous examination of the tumour-capsule interface/surrounding-tissue interface, such as the protocol proposed by Seethala *et al*.^5^. The diagnosis of NIFTP in our study occurred in a prevalent female population (74%) with an average age of 55 years, 10 years older than the multicentric series of Nikiforov’s that enrolled cases with diameter larger than 1 cm and were generally unifocal^2^. Effectively, these characteristics are related with an earlier age of diagnosis; moreover, the reference study on NIFTP started from second-level international institutions that selectively enroll neoplastic patients for the surgical treatment with higher aggressiveness or higher tumour-stage. On the other hand, our working group planned a monocentric study from a first-level general hospital that is primarily focused on benign thyroid pathology in patients with a more advanced age. In particular, a direct consequence was the unexpected high rate of *not-solitary* NIFTPs (60.1%). This percentage differs from other previously reported data that associated a NIFTP diagnosis to a “single nodule” clinical presentation; however more recent papers revealed more similar percentages with goiter or chronic pseudonodular lymphocitic thyroiditis as concurrent diseases^8^. We also recalled that a significant proportion of them were incidental (29% of multinodular cases) and a significant fraction subcentimetric (41%). The treatment of incidental thyroid tumours is still debated: some authors suggest a conservative surgical approach while Maturo *et al*. suggest total thyroidectomy because these tumours are commonly multifocal and/or bilateral and show occult lymph node metastasis^26,27^. In their study, Gartland and Lubitz reported that lobectomy and total thyroidectomy yield comparable oncologic outcomes for PTC measuring 1 to 4 cm, including cases of NIFTP^28–30^. Concerning the behaviour of the so-called *micro-NIFTP* (with a diameter of less than 1 cm), the literature showed a morphology and an indolent behavior very similar to that of the larger NIFTPs^3,31–33^. Micro-NIFTPs may occur as incidental findings in thyroidectomies performed for struma or other pathological conditions. On the contrary, for very-large NIFTPs (>4 cm) it was questioned whether the only surgical treatment without completion with radioiodine and TSH suppression therapy was a possible option, since the American Thyroid association guidelines suggests the combination of radioiodine therapy and surgical treatment^34^. Xu *et al*. reported the absence of disease recurrence in 37 patients with NIFTP over 4 cm in size that were not treated with radioiodine, and this result was confirmed by a study by Rosario *et al*.^3,33,35^. Therefore, NIFTPs of diameter greater than 4 cm are also characterised by having an indolent course after surgical treatment alone, which can therefore be the treatment of choice without the need to add a therapy with radioiodine and/or suppression of TSH. In our experience, we didn’t observe any recurrence or disease progression in both pre-2016 radioiodine-treated pT2/pT3 EFVPTCs and post-2016 radioiodine-free NIFTPs.

**Figure 3:**
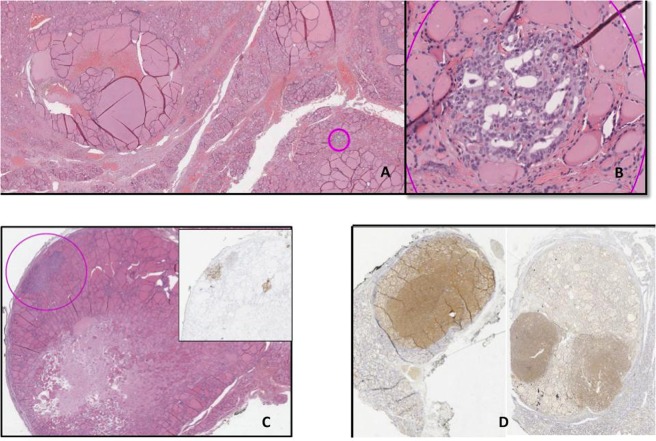
(**A**,**B**) Incipient (micro)NIFTP in the context con multinodular goiter (pink circle H&E, 4 and 20x). (**C**,**D**) The heterogeneous spectrum of multifocal NIFTP; (**C**) sprinkling phenomenon in multinodular goiter with HBME-1 correspective IHC staining (H&E 4x); (**D**) multifocal-bilateral NRAS q61r positive NIFTPs (IHC staining, 4x).

Comparing cases that had preoperative FNA performed on target lesions Vs incidental NIFTPs, diameters of these two subgroups showed a statistically significant difference (p-value 0.039). In a previous study of our group that investigated the problem of incidental diagnoses in a PTCs series, the frequency of *incidentalomas* accounted for 45.2%^25^. Accidental diagnoses were more associated with size and a multinodular clinical presentation; both small (difficult to detect) and large (so-called prevailing) nodules were possible pitfalls^25^. In wide lesions (>4 cm) FNA- sampling only occurs in a limited area of the tumour, whilst the classical NIFTP diagnostic nuclear alterations may be present only in partial areas *(sprinkling)*. In the pre-NIFTP era, Vanzati *et al*. have described cases of Follicular Variant of PTC (FVPTC) that, although at a lower magnification might appear as follicular adenomas (FA) or hyperplastic nodules, at high magnification presented microfollicles with PTC nuclear characteristics^10^. These modifications could be correlated with a genetic multifocality that is developing from a hyperplastic nodule or from a FA^10^. Compared to the multifocality parameter, 14.7% of our NIFTPs were plurifocal, as recently described^6,8^; 70% of these were bilateral. Multifocal NIFTPs are similar in appearance to multifocal classical PTC but, whilst some multifocal PTCs may derive from an intrathyroid lymphatic diffusion, this could not be true for NIFTP cases given that, by definition, they cannot exhibit lymphatic invasion (Fig. 3)^9^. Thompson *et al*. showed that this did not result in disease recurrence in patients treated and undergoing follow-up^6^. Schmitt recently underlined that multifocality and bilaterality are part of the spectrum of NIFTP, so active surveillance of the contralateral lobe should be considered when a conservative surgical approach is chosen^8^. Data regarding the clonal relationship between different synchronous intrathyroid NIFTP foci is poor and this aspect could be studied in future genetic projects. The decrease in terms of prevalence of PTC undoubtedly affects the ROM associated with diagnostic cytological classes of FNAs^3,7,11–16^. In our series, the majority of NIFTPs were classified at FNA in the indeterminate (TIR3) category (50%) and the second most frequent diagnostic class was suspicious (TIR4, 32%). The remaining cases were distributed in the benign and malignant diagnostic categories, with a slight prevalence of the benign category compared to malignancy (14% Vs 5%).The more recent updating of the italian SIAPEC system for reporting thyroid pathology, encourage to use the sub-category Tir 3b with reference to NS2/3 when referring to the possibility of a NIFTP on cytological material (the equivalent category could be THY3F for the British classification)^22^. This would facilitate appropriate programming of surgical therapy. It should be noted that the percentages of NIFTP distribution within the indeterminate cytological diagnostic categories reported in the various literature studies are very variable: from 13% to 57% for the atypical cells of undetermined significance -AUS- /follicular lesions of undetermined significance -FLUS- category of the Bethesda System for Reporting Thyroid Cytopathology, from 13% to 82% for the follicular neoplasms (SFN/FN) category and from 13% to 35% for the suspicious of malignancy^7,12–14,21,36,37^. This variability is attributable to several factors, such as the lack of safe cytological criteria with reliability and high inter/intra-observer reproducibility, the lack of a defined diagnostic class in which to insert the NIFTP and the number of NIFTPs that are diagnosed at an institution. In fact, the comparison of our case study with that of another Italian centre that took into account a similar number of cases (43 versus 37), and with the review of Bongiovanni *et al*., did not show statistically significant differences (p-value equal to 0.749 and 0.567 respectively)^16,21^. In this sense, there is a general agreement that it is not possible (nor it is desirable) to diagnose NIFTP on FNAs. The introduction of a semi-quantitative parameter, such as the NS may only allow to propose a suspicious of NIFTP on cytological preparations. In our series the number of cases with NS 2–3 were lower on cytological smears, since, in histology, a NS 1 is excluded by definition (p-value 0.002). Subsequently, NIFTP are frequently allocated to the indeterminate for malignancy cytological class (50%) with a surgical approach being selected, that seems to be justified by the NS trend. Brandler *et al*. compared the cytological characteristics of FAs, NIFTPs and PTCs on cytological smears, highlighting how, at this level, NIFTPs show characteristics in common with both the FA (microfollicular pattern) and the PTC (nuclear alterations), that are potentially indicative for this lesion if considered contemporaneously^12,38^. However, the diagnosis of NIFTP can currently only occur on histological sections because of the need for a scrupulous evaluation of the tumour capsule to rule out its invasion^39^.

## Conclusions

The NIFTP era is changing the approach to thyroid histopathology; physicians should envision a NIFTP diagnosis not only for solitary nodules, having important consequences both in the ecographic triage and in the pathological examination. Multifocality and bilaterality are part of the spectrum of NIFTPs, that can arise in a multinodular background with variable sizes from microscopic lesions to very large ones. In this sense, a close monitoring of the contralateral lobe is justified in patients that are not submitted to total thyroidectomy. Moreover, the heterogeneity of PTC-like nuclear features is a significant factor that may affect histopathology. The possible coexistence of two lesions and the so-called *sprinkling* phenomenon should be considered carefully. Finally, although a NIFTP diagnosis may be incidental (especially in a multinodular goiter presentation, where FNA is performed on a different target), cytopathological criteria such as the NS evaluation may help the pathologists in including NIFTP in the differential diagnosis of indeterminate cases in the preoperative setting and to more appropriately select the correct treatment.

## References

[1] Lloyd, R. V., Osamura, R. Y., Kioppel, G. & Rosai, J. International Agency for Research on Cancer. WHO Classification of Tumours of Endocrine Organs. *IARC Who Classification of Tumour*s, Lyon (2017).

[2] Nikiforov YE (2016). Nomenclature Revision for Encapsulated Follicular Variant of Papillary Thyroid Carcinoma: A Paradigm Shift to Reduce Overtreatment of Indolent Tumors. JAMA Oncol.

[3] Tallini G, Tuttle RM, Ghossein RA (2017). The History of the Follicular Variant of Papillary Thyroid Carcinoma. J Clin Endocrinol Metab..

[4] Hung YP, Barletta JA (2017). A user’s guide to non-invasive follicular thyroid neoplasm with papillary-like nuclear features (NIFTP). Histopathology.

[5] Seethala RR (2018). Noninvasive follicular thyroid neoplasm with papillary-like nuclear features: a review for pathologists. Mod Pathol.

[6] Thompson LD (2016). Ninety-four cases of encapsulated follicular variant of papillary thyroid carcinoma: A name change to Noninvasive Follicular Thyroid Neoplasm with Papillary-like Nuclear Features would help prevent overtreatment. Mod Pathol.

[7] Amendoeira I, Maia T, Sobrinho-Simões M (2018). Non-invasive follicular thyroid neoplasm with papillary-like nuclear features (NIFTP): impact on the reclassification of thyroid nodules. Endocr Relat Cancer.

[8] Canberk Sule, Montezuma Diana, Taştekin Ebru, Grangeia Diana, Demirhas Mehmet Polat, Akbas Meryem, Tokat Fatma, Ince Umit, Soares Paula, Schmitt Fernando (2019). “The other side of the coin”: understanding noninvasive follicular tumor with papillary-like nuclear features in unifocal and multifocal settings. Human Pathology.

[9] LiVolsi VA, Baloch ZW (2017). Coming to Terms with Diagnosis “Non-Invasive Follicular Neoplasm with Papillary Like Nuclear Features (NIFTP)’: Practice Changer in Endocrine Pathology. Journal of Basic and Clinical Medicine.

[10] Vanzati A, Mercalli F, Rosai J (2013). The “Sprinkling” Sign in the Follicular Variant of Papillary Thyroid Carcinoma: A Clue to the Recognition of This Entity. Arch Pathol Lab Med.

[11] Jug R, Jiang X (2017). Noninvasive Follicular Thyroid Neoplasm with Papillary-Like Nuclear Features: An Evidence-Based Nomenclature Change. Patholog Res Int.

[12] Brandler TC (2017). Can noninvasive follicular thyroid neoplasm with papillary-like nuclear features be distinguished from classic papillary thyroid carcinoma and follicular adenomas by fine-needle aspiration?. Cancer Cytopathol.

[13] Faquin WC (2015). Impact of reclassifying noninvasive follicular variant of papillary thyroid carcinoma on the risk of malignancy in The Bethesda System for Reporting Thyroid Cytopathology. Cancer Cytopathol.

[14] Strickland KC (2015). The Impact of Noninvasive Follicular Variant of Papillary Thyroid Carcinoma on Rates of Malignancy for Fine-Needle Aspiration Diagnostic Categories. Thyroid.

[15] Zhou H (2018). Noninvasive follicular thyroid neoplasm with papillary-like nuclear features (NIFTP): Implications for the risk of malignancy (ROM) in the Bethesda System for Reporting Thyroid Cytopathology (TBSRTC). Cancer Cytopathol.

[16] Bongiovanni M, Giovanella L, Romanelli F, Trimboli P (2019). Cytological Diagnoses Associated with Noninvasive Follicular Thyroid Neoplasms with Papillary-Like Nuclear Features According to the Bethesda System for Reporting Thyroid Cytopathology: A Systematic Review and Meta-Analysis. Thyroid..

[17] Nardi F (2014). Italian consensus for the classification and reporting of thyroid cytology. J Endocrinol Invest.

[18] Bongiovanni M (2012). Comparison of 5-tiered and 6-tiered diagnostic systems for the reporting of thyroid cytopathology: a multi-institutional study. Cancer Cytopathol.

[19] Ali, S. Z. & Cibas, E. S. The Bethesda System for Reporting Thyroid Cytopathology: Definitions, Criteria, and Explanatory Notes. Second edition. Springer (2017).

[20] Gharib H (2016). American association of clinical endocrinologists, american college of endocrinology, and associazione medici endocrinologi medical guidelines for clinical practice for the diagnosis and management of thyroid nodules–2016 update. Endocr Pract.

[21] Bizzarro T (2016). Young investigator challenge: The morphologic analysis of noninvasive follicular thyroid neoplasm with papillary-like nuclear features on liquid-based cytology: Some insights into their identification. Cancer Cytopathol.

[22] Rossi ED (2019). Noninvasive Follicular Thyroid Neoplasm with Papillary-Like Nuclear Features (NIFTP): Update and Diagnostic Considerations-a Review. Endocr Pathol.

[23] Burman KD, Wartofsky L (2016). Thyroid Nodules. N Engl J Med.

[24] Brito JP (2014). The accuracy of thyroid nodule ultrasound to predict thyroid cancer: systematic review and meta-analysis. J Clin Endocrinol Metab.

[25] Pagni F (2014). Incidental Papillary Thyroid Carcinoma: Diagnostic Findings in a Series of 287 Carcinomas. Endocr Pathol.

[26] Pigac B, Masic S, Hutinec Z, Masic V (2018). Rare Occurrence of Incidental Finding of Noninvasive Follicular Thyroid Neoplasm With Papillary-Like Nuclear Features in Hürthle Cell Adenoma. Mediev Archaeol.

[27] Maturo A (2017). Incidental thyroid carcinomas. A retrospective study. Giornale di Chirurgia - Journal of Surgery.

[28] Gartland RM, Lubitz CC (2018). Impact of Extent of Surgery on Tumor Recurrence and Survival for Papillary Thyroid Cancer Patients. Ann Surg Oncol.

[29] Rosario PW (2019). Impact of Noninvasive Follicular Thyroid Neoplasm with Papillary-Like Nuclear Features (NIFTP) on the Outcomes of Lobectomy. Ann Surg Oncol..

[30] Gartland RM, Lubitz CC (2019). Reply to “Impact of Noninvasive Follicular Thyroid Neoplasm with Papillary-Like Nuclear Features (NIFTP) on the Outcomes of Lobectomy. Ann Surg Oncol..

[31] Xu B (2018). Should subcentimeter non-invasive encapsulated, follicular variant of papillary thyroid carcinoma be included in the noninvasive follicular thyroid neoplasm with papillary-like nuclear features category?. Endocrine.

[32] Rosario PW (2018). Subcentimetre non-invasive follicular thyroid neoplasm with papillary-like nuclear features (NIFTP). Histopathology.

[33] Rosario PW (2017). Long-Term Outcomes of Patients with Noninvasive Follicular Thyroid Neoplasm with Papillary-Like Nuclear Features (NIFTP) ≥4 cm Treated Without Radioactive Iodine. Endocr Pathol.

[34] Haugen BR (2016). 2015 American Thyroid Association Management Guidelines for Adult Patients with Thyroid Nodules and Differentiated Thyroid Cancer: The American Thyroid Association Guidelines Task Force on Thyroid Nodules and Differentiated Thyroid Cancer. Thyroid.

[35] Xu B (2017). Outcome of Large Noninvasive Follicular Thyroid Neoplasm with Papillary-Like Nuclear Features. Thyroid.

[36] Lee SE (2017). Molecular Profiling of Papillary Thyroid Carcinoma in Korea with a High Prevalence of BRAFV600E Mutation. Thyroid.

[37] Maletta F (2016). Cytological features of “noninvasive follicular thyroid neoplasm with papillary-like nuclear features” and their correlation with tumor histology. Hum Pathol.

[38] Brandler TC (2019). Noninvasive follicular thyroid neoplasm with papillary-like nuclear features: An interobserver study of key cytomorphological features from a large academic medical centre. Cytopathology.

[39] Trabzonlu L, Paksoy N (2018). Cytomorphological Analysis of Thyroid Nodules Diagnosed as Follicular Variant of Papillary Thyroid Carcinoma: a Fine Needle Aspiration Study of Diagnostic Clues in 42 Cases and the Impact of Using Bethesda System in Reporting-an Institutional Experience. Endocr Pathol.

